# Acute kidney injury due to rhabdomyolysis and renal replacement therapy: a critical review

**DOI:** 10.1186/cc13897

**Published:** 2014-05-28

**Authors:** Nadezda Petejova, Arnost Martinek

**Affiliations:** 1 Department of Internal Medicine, University Hospital Ostrava, 17 listopadu 1790, 708 52 Ostrava, Czech Republic

## Abstract

Rhabdomyolysis, a clinical syndrome caused by damage to skeletal muscle and release of its breakdown products into the circulation, can be followed by acute kidney injury (AKI) as a severe complication. The belief that the AKI is triggered by myoglobin as the toxin responsible appears to be oversimplified. Better knowledge of the pathophysiology of rhabdomyolysis and following AKI could widen treatment options, leading to preservation of the kidney: the decision to initiate renal replacement therapy in clinical practice should not be made on the basis of the myoglobin or creatine phosphokinase serum concentrations.

## Introduction

Rhabdomyolysis (RM) is a clinical syndrome characterized by injury to skeletal muscle fibers with disruption and release of their contents into the circulation. Myoglobin, creatine phosphokinase (CK) and lactate dehydrogenase are the most important substances for indicating muscle damage [1].

## Brief history

The history of RM goes back to the Second World War in 1941 when the condition was described for the first time. The London Blitz was the sustained strategic bombing of many cities in the United Kingdom and the ensuing crush injuries led to typical symptoms of RM [2]. Today, we know the causes of RM are legion and include trauma, drugs such as statins, infections, toxins, extreme physical exertion, temperature extremes, hereditary and acquired metabolic myopathies [3].

## Clinical symptoms

The clinical symptoms of RM are well known: myalgia, weakness and swelling involving injured muscles, usually associated with myoglobinuria. The clinical symptoms might include nonspecific symptoms such as fever, nausea, dyspepsia and/or vomiting. Mild and subclinical cases of RM, called in clinical practice myopathies, are typically characterized by elevated serum CK and myalgias [4].

The severity of RM escalates from myoglobinuria, which can result in acute kidney injury (AKI), to other severe systemic complications such as disseminated intravascular coagulopathy and acute compartment syndrome from swelling muscle, and reduced macrocirculation and microcirculation of injured limbs. Extracted fluid from the circulation into the swollen muscle groups leads to hypotension and shock. Typical metabolic alterations accompanying RM are hyperkalemia, metabolic acidosis, hypocalcemia or hypercalcemia, hyperuricemia, hyponatremia and hyperphosphatemia with possible cardiac dysrhythmias [5,6]. AKI due to rhabdomyolysis occurs in 13 to 50% of all cases [7].

## Etiology

As already mentioned, the development of RM is associated with a large number of conditions and pathological disorders. Medical research, reviews, studies and case reports describe different possible causes of RM (Table 1) [3,8,9].

**Table 1:** Etiology of rhabdomyolysis and myopathies

**Acquired**	**Hereditary**
Extreme physical activity	Metabolic myopathies caused by disorders of:
Influence of extreme temperatures	Fatty acid oxidation
Metabolic disorders of water and salts	Mitochondrial metabolism
Trauma and crush syndrome	Glycolysis/glycogenolysis
Vascular ischemia	Purine nucleotide cycle
Influence of drugs	Pentose phosphate pathway
Infections, sepsis	
Toxins	
Malignant hyperthermia	
Endocrine disorders	
Electrical current	

### Pathophysiology of rhabdomyolysis and following acute kidney injury

Under physiological conditions, skeletal muscle cell contraction requires a nervous impulse originating in a voluntary process. The nervous impulse is then transferred to a thin muscle cell membrane called the sarcolemma. The sarcolemma is a physical barrier and mediator between cell and external signals. In healthy myocytes, the sarcolemma contains different pumps for regulating the process of cellular electrochemical gradients [10]. The most important is Na-K-ATP-ase for sodium and potassium exchange. Under normal conditions, sodium ions are actively excluded from the muscle cell and potassium ions are allowed passage. This process is energy dependent and builds on calcium removal in Na/Ca by changing the intracellular electrical gradient during active removal of sodium. Both processes depend on ATP as a source of energy [11,12].

The sarcoplasm is the specialized cytoplasm of the muscle cell that contains the usual subcellular elements along with Golgi apparatus, myofibrils, a modified endoplasmic reticulum known as the sarcoplasmic reticulum, myoglobin and mitochondria. The primary function of the sarcoplasmic reticulum is to store calcium, which is released by muscular contraction. The most serious consequence of RM is ATP depletion, resulting in membrane cell pump dysfunction. The extrusion of sodium is impaired and the efflux of calcium from the cell is impaired [13]. If a high concentration of calcium persists in the sarcoplasm this activates cytolytic enzymes such as hydroxylases, proteases, nucleases and many others. The following impairment of cell organelles, especially of mitochondria, leads to progressive decrease in ATP, the production of free oxygen radicals and cell damage [12]. The result of cell impairment is release of potassium, phosphates, myoglobin, CK, lactate dehydrogenase and aldolase into the blood circulation with typical clinical presentation of RM.

### Acute kidney injury is one of the most severe complications of rhabdomyolysis

The pathophysiology of RM-induced AKI is believed to be triggered by myoglobin as the toxin causing renal dysfunction [14]. This claim is given substance from studies in animal models of glycerol-induced AKI. Intramuscular injection of glycerol in the rabbit induces a model of AKI at a dose of 10 mg/kg that resembles the AKI caused by massive release of myoglobin in crush syndrome in humans [15]. Glycerol-induced AKI is characterized by myoglobinuria, tubular necrosis and renal vasoconstriction [16]. The most important role in glycerol-induced nephrotoxicity has been attributed to reactive oxygen metabolites (reactive oxygen species), in particular the hydroxyl radical (OH^.^), the same cause as for myoglobin-induced AKI [17].

Myoglobin is an oxygen and iron binding protein with a molecular weight of 17,500 Da. Myoglobin is found in the muscle tissue of vertebrates, has a higher affinity for oxygen than hemoglobin and assists myocytes to acquire energy. Myoglobin can be detected in urine in small concentrations <5 μg/l, but meets the diagnostic criteria for myoglobinuria at concentrations >20 μg/l [9].

Myoglobin – which may undergo reabsorption from the glomerular filtrate, is catabolized within proximal tubule cells and is easily filtered through the glomerular basement membrane – has been recognized as playing a part in the development of AKI in the setting of myoglobinuria. The clinical study by Gburek and colleagues demonstrated that renal uptake of myoglobin is mediated by the endocytic receptors, megalin and cubilin [18]. The same membrane cell receptors play an important role in nephrotoxicity; for example, those of antibiotics.

**Figure 1:**
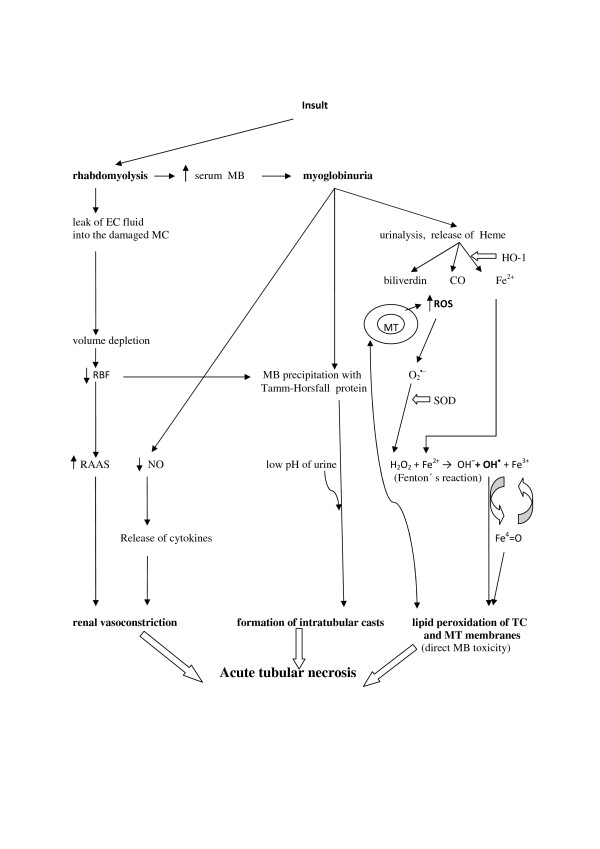
**Pathophysiology of rhabdomyolysis-induced acute kidney injury.** CO, carbon monoxide; EC, extracellular; Fe^2+^, ferrous iron; Fe^3+^, ferric iron; Fe^4^ = O, ferryl iron; HO-1, heme oxygenase-1; H_2_O_2_, hydrogen peroxide; MB, myoglobin; MC, muscle cell; MT, mitochondria; NO, nitric oxide; OH^−^, hydroxyl anion; O_2_^•–^, superoxide radical; OH^•^, hydroxyl radical, RAAS, renin–angiotensin–aldosterone system; RBF, renal blood flow; ROS, reactive oxygen species; SOD, superoxide dismutase; TC, tubular cell.

The three different mechanisms of renal toxicity by myoglobin are usually reported as renal vasoconstriction, formation of intratubular casts and the direct toxicity of myoglobin to kidney tubular cells [19-27] (Figure 1).

Renal vasoconstriction is caused by reduced renal blood flow due to excessive leakage of extracellular fluid into the damaged muscle cells and by secondary activation of the renin–angiotensin–aldosterone system. However, a second theory favors the effect of the nitric oxide scavenging characteristics of myoglobin and release of cytokines [25,26]. The formation of intratubular casts explains the urine concentration and the following reaction of myoglobin with Tamm–Horsfall tubular protein. Further, renal vasoconstriction, the decrease in renal blood flow due to volume depletion and the low pH of urine promote this pathological process by formation of stronger and more rapid bonds between Tamm–Horsfall protein and myoglobin [12,20].

Heme released from myoglobin is, under normal conditions, degraded by the enzyme heme oxygenase-1 with marked vasodilatating effect. Heme oxygenase-1 is upregulated in proximal tubular cells in response to oxidant stress and exerts cytoprotective and anti-inflammatory effects [14,21,22]. Zager and colleagues [19] studied intrarenal heme oxygenase-1 induction in response to four different experimental AKI models: glycerol, cisplatin, ischemic–reperfusion and a bilateral ureteral obstruction model. In the glycerol AKI model that best reflects kidney damage during myoglobinuric AKI, heme oxygenase-1 was detectable in plasma and the renal cortex, and these changes were associated with an approximately 10-fold increase in renal heme oxygenase-1 mRNA. With the urinary heme oxygenase-1 concentration increase in the glycerol AKI model, further increases were observed 4 and 24 hours after glycerol injection. Finally, the authors tested whether the above findings might have clinical relevance in 20 critically ill patients: one-half of the patients had AKI and one-half had no AKI. Only the AKI group had significantly elevated plasma and urinary heme oxygenase-1 concentrations, and these investigations led to the conclusion that AKI can evoke heme oxygenase-1 elevation in plasma and urine [19]. However, the whole molecular pathophysiology of myoglobin-induced AKI is based on the deleterious effects of reactive oxygen species directly on the tubular cells and their organelles.

Reactive oxygen species also play an important and protective role in the living organism against pathogens and cancer during phagocytosis and other, especially metabolic, reactions. But overproduction of reactive oxygen species may lead to damage to living cells via lipid peroxidation of fatty acids and to the production of malondialdehyde, which can cause the polymerization of protein and DNA [23]. The hydroxyl radical is the most reactive of the reactive oxygen species group and is produced by the reaction between superoxide and hydrogen peroxide catalyzed by iron in Fenton’s reaction (Figure 1).

In previous years, iron-mediated hydroxyl radical production with resultant oxidant stress was hypothesized to be the dominant pathway for heme protein nephrotoxicity [17]. However, it was later shown that Fe-mediated proximal tubular system lipid peroxidation was more hydrogen peroxide dependent than hydroxyl anion (OH^−^) dependent and that blockage of myoglobin cytotoxicity via only decreasing hydroxyl anion generation may be inadequate [24]. For myoglobin to catalyze lipid peroxidation, ferrous (Fe^2+^) myoglobin must be oxidized to the ferric (Fe^3+^) form, which leads to induced lipid peroxidation by redox cycling with ferryl (Fe^4^ = O) myoglobin. This is a highly reactive form of myoglobin, which can potently induce lipid peroxidation [27]. Redox cycling between ferric and ferryl myoglobin yields radical species that cause severe oxidative damage to the kidney [28].

This process has been shown to be pH dependent and alkaline conditions prevent myoglobin-induced lipid peroxidation by stabilizing the reactive ferryl–myoglobin complex [29,30]. Alkaline conditions stabilize the ferryl species, making myoglobin considerably less reactive towards lipids and lipid hydroperoxides [31]. The fact that RM can be considered an oxidative stress-mediated pathology also with mitochondria as the primary target, and possibly the source of reactive oxygen and nitrogen species, has been reported in a study by Plotnikov and colleagues [32]. However, the authors speculate that RM-induced kidney damage involves direct interaction of myoglobin with mitochondria possibly resulting in iron ion release from myoglobin’s heme, and this promotes the peroxidation of mitochondrial membranes [32]. This problem, however, appears to be more complicated.

In summary, better knowledge of the pathophysiology can optimize prevention and treatment measures in cases of RM kidney injury.

## Diagnosis

In typical clinical conditions, patients with RM experience muscular weakness, myalgia, swelling, tenderness or stiffness and dark brown urine [1]. Correct diagnosis is the most important step to initiating proper treatment. The clinical and laboratory diagnostics summarized in Table 2 are the basic approach in differential diagnosis.

**Table 2:** Diagnosis of rhabdomyolysis and following acute kidney injury

**Clinical presentation**
Muscular weakness, myalgia, swelling, tenderness, stiffness
Fever, feelings of nausea, vomiting, tachycardia
Oligoanuria or anuria in connection with renal damage or in the presence of volume depletion
Signs of the underlying disease
**Laboratory findings**
Serum: creatinine, urea nitrogen, creatine phosphokinase, myoglobin, ions (potassium, phosphorus, calcium), lactate dehydrogenase, transaminases, acid–base balance
Urine: myoglobin or positive dipstick test without any erythrocytes

Serum myoglobin is normally bound to plasma globulins such as haptoglobin and α_2_-globulin and has a rapid renal clearance to maintain a low plasma concentration of 3 μg/l [33]. Radioimmunoassays or imunolatex, imunoturbidimetric methods can detect myoglobin in plasma or urine. Normal serum levels are 30 to 80 μg/l and normal urine levels are 3 to 20 μg/l [3]. After the development of RM, free serum myoglobin increases due to exceeding the binding capacity of plasma globulins and then kidney filtrate appears in the urine which contributes to the brownish (tea) urine color. Furthermore, urine myoglobin concentrations are normally measured to assess RM; surprisingly, one *in vitro* study observed that low pH is not by itself a cause of urine myoglobin instability. The extent of instability depended not only on urine pH and temperature but also on unidentified urinary factors and initial urinary myoglobin concentrations [34]. Another way to diagnose myoglobinuria is a positive test for the presence of blood in urine without finding erythrocytes.

Serum levels of CK correlate with the severity of RM but less so with myoglobinuric AKI. Normal serum levels are 0.15 to 3.24 μkat/l or 9 to 194 U/l in men and 0.15 to 2.85 μkat/l or 9 to 171 U/l in women. To predict AKI following RM, the clinician needs a better marker than serum CK, which is routinely used as a marker in the assessment of these disorders. Very important findings about the use of myoglobin as a marker and predictor in AKI were described by Premru and colleagues [35]. The authors investigated and restrospectively analyzed the incidence of myoglobin-induced AKI (serum creatinine >200 μmol/l) and the need for hemodialysis in 484 patients with suspected RM. The median peak myoglobin was 7,163 μg/l. The incidence of myoglobin-induced AKI was significantly higher (64.9%) in patients with a peak serum myoglobin >15,000 μg/l (*P* <0.01). Most of these patients needed treatment with hemodialysis (28%). Myoglobin levels >15,000 μg/l were most significantly related to the development of AKI and the need for hemodialysis. Based on these results, serum myoglobin was recommended as a valuable early predictor and marker of RM and myoglobinuric AKI [35].

In another retrospective observational cohort study, El-Abdellati and colleagues studied CK, serum myoglobin and urinary myoglobin as markers of RM and AKI in 1,769 adult patients. The results for the best cutoff values for prediction of AKI were CK >773 U/l, serum myoglobin >368 μg/l and urine myoglobin >38 μg/l, respectively [36].

## Conservative measures in rhabdomyolysis to prevent acute kidney injury

**Figure 2:**
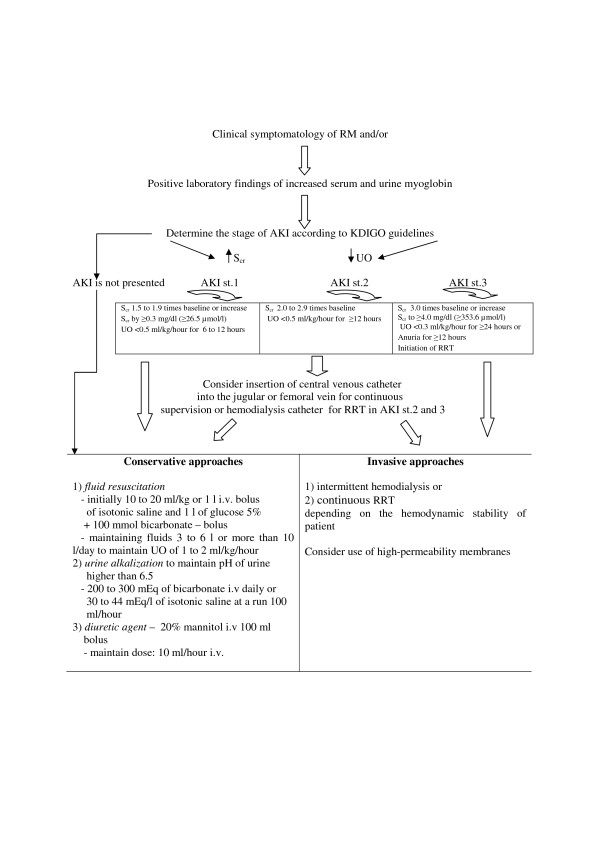
**Therapeutic approaches in rhabdomyolysis for prevention and treatment of acute kidney injury.** AKI, acute kidney injury; KDIGO, Kidney Disease Improving Global Outcomes; RM, rhabdomyolysis; RRT, renal replacement therapy; S_cr_, serum creatinine; UO, urine output.

The first step in medical intervention is usually treatment of underlying disease. In the case of preserved diuresis in the setting of RM, we must initiate conservative measures, which usually include massive hydration, use of mannitol, urine alkalization and forced diuresis [25,37-39] (Figure 2).

Early and aggressive fluid resuscitation to restore renal perfusion and increase the urine flow rate is agreed on as the main intervention for preventing and treating AKI [6]. Fluid resuscitation with crystalloid solutions is the ubiquitous intervention in critical care medicine [40]. One caveat, however, is that these therapeutic measures are not useful in the context of severe oliguria or anuria and may lead to interstitial and pulmonary edema. Clinicians have to be careful about oliguria which is a normal response to hypovolemia and should not be used solely as a trigger or end point for fluid resuscitation, particularly in the post-resuscitation period [41]. Further, while aggressive volume resuscitation may preserve cardiac output and renal perfusion pressure, in the presence of oliguria it is an independent predictor for developing secondary abdominal compartment syndrome with decreased renal perfusion pressure or can lead to acute respiratory distress syndrome [42].

### Diuretic agent – mannitol

Mannitol is an osmotic agent that attracts the fluid of the interstitial space and so may reduce muscular swelling. As a diuretic agent, mannitol prevents intrarenal heme pigment trapping, decreasing cast formation. Mannitol can increase renal blood flow and glomerular filtration. Several studies have highlighted its hydroxyl anion-scavenging effect, although in an experimental study Zager and colleagues concluded that its protective influence is probably more due to a diuretic than to antioxidant effect [43]. Bragadottir and colleagues [44] studied the effects of mannitol on renal blood flow, the glomerular filtration rate, renal oxygen consumption and extraction in 11 postoperative cardiac patients with AKI. In all patients, a bolus dose of mannitol 225 mg/kg was given, followed by an infusion at a rate of 75 mg/kg/hour for two 30-minute periods. The authors reported that mannitol treatment in these cases increased urine flow by 61% (*P* <0.001), induced renal vasodilation and redistributed systemic blood flow to the kidney. In addition, mannitol does not affect the filtration fraction or renal oxygenation [44].

There are some controversial views on post-traumatic RM, where the recommendation is to re-evaluate the standards of therapy with bicarbonate and mannitol because this combination does not prevent renal failure, dialysis or mortality in patients with CK levels >5,000 U/l [45]. Knowledge of the timing of adequate hydration in severe post-traumatic patients would be valuable.

### Antioxidant therapy

Based on the pathophysiology of myoglobinuric AKI, we can predict protective effects of antioxidative therapy by inhibition of lipid peroxidation of the proximal tubular cells and redox cycling between ferric and ferryl myoglobin [28]. Acetaminophen, which inhibits hemoprotein-catalyzed lipid peroxidation, is one of several investigated drugs that attenuate RM-induced AKI. Acetaminophen inhibits prostaglandin hydrogen synthase by reducing the protoporphyrin radical cation and blocking formation of the catalytic tyrosyl radical [46,47]. However, one experimental *in vitro* study showed that its administration is necessary after RM to achieve the desired outcome in blocking lipid peroxidation [28].

## Renal replacement therapy

The first factor we need to recognize is that the greatest filter for removing myoglobin is the kidney, and in critical care nephrology there is no preventive kidney replacement therapy. However, the kidneys need a perfusion pressure and fluid volume to help them eliminate the toxin. The initiation of renal replacement therapy in clinical practice should not be managed by the myoglobin or CK serum concentration but by the status of renal impairment, with complications such as life-threatening hyperkalemia, hypercalcemia, hyperazotemia, anuria or hyperhydration without response to diuretic therapy. For better orientation to the requirements of renal replacement therapy initialization in AKI on critical care, we can use the Kidney Disease Improving Global Outcomes guidelines. These guidelines include a comprehensive therapeutic approach for management of AKI (Figure 2) [39,48]. Renal replacement therapies are mostly efficient in cases of RM-induced AKI, but they are extracorporeal circuits with many potential complications. However, in clinical practice it is very important to take into account all coincident factors in the patient’s illness and to individualize treatment if necessary.

The possibility of using a method of renal replacement therapy, either intermittent hemodialysis or continuous kidney replacement methods in the case of RM, has been investigated in several studies [49-51]. Plasmapheresis has also been used with described success. When we proceed to treat patients with these procedures, we must take into account that myoglobin has a molecular mass of 17 kDa and is poorly removed from circulation using conventional extracorporeal techniques. Intermittent hemodialysis is mostly mandated by renal or metabolic indications and preventive extracorporeal elimination is not recommended. Sorrentino and colleagues [52] reported the effective removal of myoglobin by extended dialysis performed using a single-pass batch dialysis system and a polysulphone high-flux dialyzer (surface area 1.8 m^2^), allowing elimination of substances with a molecular weight of up to 30 kDa. In six patients with myoglobinuric AKI, a median myoglobin clearance of 90.5 ml/minute (range 52.4 to 126.3 ml/minute) was achieved, resulting in a median myoglobin removal per treatment hour of 0.54 g (range 0.15 to 2.21 g) [52].

Myoglobin clearance by super high-flux hemofiltration in a 53-year-old female suffering from RM and AKI was investigated by Naka and colleagues [53]. Continuous venovenous hemofiltration was performed with a high-permeability membrane (cutoff point 100 kDa) at 2 to 4 l/hour ultrafiltration in an attempt to clear myoglobin. The sieving coefficient was 68 to 72%, myoglobin clearance was up to 56.4 l/day and the amount of myoglobin removed was 4.4 to 5.1 g/day [53]. The effect of high cutoff membrane hemodiafiltration on myoglobin removal was investigated in six patients with myoglobinuric AKI using a 45 kDa cutoff hemofilter with a surface area 2.1 m^2^. Postdilutional fluid substitution was 2 to 3 l/hour, resulting in a mean myoglobin clearance of 81 ml/minute (range 42 to 131 ml/minute). The reduction ratio ranged from 62 to 89% [54].

The use of plasmapheresis to remove serum myoglobin sounds controversial but successful therapy of RM induced by statins was reported by Swaroop and colleagues [55]. The plasmapheresis was performed in addition to hemodialysis daily for 5 days. The effect of hemodialysis alone is questionable, and the authors did not describe the type of hemodialysis or membrane used [55]. The undeniable fact is the obligation to treat underlying disease that led to RM. If we do not eliminate the cause of the RM – especially in trauma, infectious disorders and septic disorders – the removal of myoglobin is only a supportive measure for increasing its clearance in the case of AKI. However, inefficient removal of myoglobin also results in a persistent high circulating level of the molecule with kidney damage and delay in renal function recovery [56]. From all these data, the effect of high-permeability membranes on eliminating circulating myoglobin has been demonstrated but care must be taken to prevent unwanted albumin loss. It is questionable whether a percentage of myoglobin clearance is not hepatic because of a decrease in serum myoglobin in patients with oligoanuric AKI. The recommended most useful mode of renal replacement therapy used to be hemofiltration, but in recent years we use high-permeability membranes in daily clinical practice for continuous venovenous hemodialysis without undesirable decrease in albumin levels. The molecular weight of albumin is 67 kDa and high-permeability membranes with a cutoff value <67 kDa in a predominantly diffusion type of elimination can be a prospective measure for the supplementary treatment of RM if necessary.

## Conclusions

This review provides a comprehensive view on AKI induced by RM. Thorough knowledge of the pathophysiology will lead to new approaches for diagnosis and treatment, leading to the preservation of the kidney. Renal replacement methods have a supportive role but they are not the first line of treatment for AKI-induced RM, especially in cases of preserved diuresis. The kidney is a miraculous organ but it can be overwhelmed if the threshold is exceeded. We should try to preserve kidney function where possible by looking at the whole picture.

## Abbreviations

AKI: Acute kidney injury; CK: Creatine phosphokinase; RM: Rhabdomyolysis.

## Competing interests

The authors declare that they have no competing interests.

## References

[B1] Bagley WH Yang H Shah KH Rhabdomyolysis Intern Emerg Med 2007 2 210 218 10.1007/s11739-007-0060-8 17909702

[B2] Beall D Bywaters EG Belsey RH Miles JA Crush injury with renal failure Br Med J 1941 1 432 434 10.1136/bmj.1.4185.432 20783578 PMC2161708

[B3] Vanholder R Sever MS Erek E Lameire N Rhabdomyolysis J Am Soc Nephrol 2000 11 1553 1561 10906171 10.1681/ASN.V1181553

[B4] Owczarek J Jasińska M Orszulak-Michalak D Drug-induced myopathies. An overview of the possible mechanisms Pharmacol Res 2005 57 23 34 15849374

[B5] Cervellin G Comelli I Lippi G Rhabdomyolysis: historical background, clinical, diagnostic and therapeutic features Clin Chem Lab Med 2010 48 749 756 20298139 10.1515/CCLM.2010.151

[B6] Zimmerman JL Shen MC Rhabdomyolysis Chest 2013 144 1058 1065 10.1378/chest.12-2016 24008958

[B7] Zager RA Studies of mechanisms and protective maneuvers in myoglobinuric acute renal injury Lab Invest 1989 60 619 629 2716281

[B8] Lima RS da Silva Junior GB Liborio AB Daher Ede F Acute kidney injury due to rhabdomyolysis Saudi J Kidney Dis Transpl 2008 19 721 729 18711286

[B9] David WS Myoglobinuria Neurol Clin 2000 18 215 243 10.1016/S0733-8619(05)70187-0 10658177

[B10] Hirsch NP Neuromuscular junction in health and disease Br J Anaesth 2007 99 132 138 10.1093/bja/aem144 17573397

[B11] Knochel JP Mechanisms of rhabdomyolysis Curr Opin Rheumatol 1993 5 725 731 10.1097/00002281-199305060-00006 8117534

[B12] Zager RA Rhabdomyolysis and myohemoglobinuric acute renal failure Kidney Int 1996 49 314 326 10.1038/ki.1996.48 8821813

[B13] Better OS Abassi Z Rubinstein I Marom S Winaver Y Silberman M The mechanism of muscle injury in the crush syndrome: ischemic versus pressure-stretch myopathy Miner Electrolyte Metab 1990 16 181 184 2277600

[B14] Nath KA Balla G Vercellotti GM Balla J Jacob HS Levitt MD Rosenberg ME Induction of heme oxygenase is a rapid, protective response in rhabdomyolysis in the rat J Clin Invest 1992 90 267 270 10.1172/JCI115847 1634613 PMC443091

[B15] Trillaud H Degrèze P Combe C Deminière C Palussière J Benderbous S Grenier N USPIO-enhanced MR imaging of glycerol-induced acute renal failure in the rabbit Magn Reson Imaging 1995 13 233 240 10.1016/0730-725X(94)00114-I 7739365

[B16] Singh AP Junemann A Muthuraman A Jaggi AS Singh N Grover K Dhawan R Animal models of acute renal failure Pharmacol Rep 2012 64 31 44 22580518 10.1016/s1734-1140(12)70728-4

[B17] Shah SV Walker PD Evidence suggesting a role for hydroxyl radical in glycerol-induced acute renal failure Am J Physiol 1988 255 F438 F443 2843051 10.1152/ajprenal.1988.255.3.F438

[B18] Gburek J Birn H Verroust PJ Goj B Jacobsen C Moestrup SK Willnow TE Christensen EI Renal uptake of myoglobin is mediated by the endocytic receptors megalin and cubilin Am J Physiol Renal Physiol 2003 285 451 458 10.1152/ajprenal.00062.2003 12724130

[B19] Zager RA Johnson AC Becker K Plasma and urinary heme oxygenase-1 in AKI J Am Soc Nephrol 2012 23 1048 1057 10.1681/ASN.2011121147 22440905 PMC3358765

[B20] Krouzecky A Matejovic M Rokyta R Novak I Rhabdomyolysis – mechanisms of origin, causes, consequences and therapy Vnitr Lek 2003 49 668 672 14518093

[B21] Agarwal A Balla J Alam J Croatt AJ Nath KA Induction of heme oxygenase in toxic renal injury: a protective role in cisplatin nephrotoxicity in the rat Kidney Int 1995 48 1298 1307 10.1038/ki.1995.414 8569092

[B22] Nath KA Heme oxygenase-1: a provenanc pathways in the kidney and other tissues Kidney Int 2006 70 432 443 16775600 10.1038/sj.ki.5001565

[B23] Závada J Multiple Organ Dysfunction Syndrome 2001 Praha: Grada Publishing s.r.o

[B24] Zager RA Foerder CA Effects of inorganic iron and myoglobin on in vitro proximal tubular lipid peroxidation and cytotoxicity J Clin Invest 1992 89 989 995 10.1172/JCI115682 1311724 PMC442948

[B25] Ronco C Bellomo R Kellum JA Critical care nephrology Myoglobin as a Toxin 2009 2 Philadelphia, PA: Saunders, Elsevier 1103 1109

[B26] Blomberg LM Blomberg MR Siegbahn PE A theoretical study of myoglobin working as a nitric oxide scavenger J Biol Inorg Chem 2004 9 923 935 10.1007/s00775-004-0585-5 15452775

[B27] Moore KP Holt SG Patel RP Svistunenko DA Zackert W Goodier D Reeder BJ Clozel M Anand R Cooper CE Morrow JD Wilson MT Darley-Usmar V Roberts LJ 2nd A causative role for redox cycling of myoglobin and its inhibition by alkalinization in the pathogenesis and treatment of rhabdomyolysis-induced renal failure J Biol Chem 1998 273 31731 31737 10.1074/jbc.273.48.31731 9822635

[B28] Boutaud O Moore KP Reeder BJ Harry D Howie AJ Wang S Carney CK Masterson TS Amin T Wright DW Wilson MT Oates JA Roberts LJ 2nd Acetaminophen inhibits hemoprotein-catalyzed lipid peroxidation and attenuates rhabdomyolysis-induced renal failure Proc Natl Acad Sci U S A 2010 107 2699 2704 10.1073/pnas.0910174107 20133658 PMC2823910

[B29] Holt S Moore K Pathogenesis of renal failure in rhabdomyolysis: the role of myoglobin Exp Nephrol 2000 8 72 76 10.1159/000020651 10729745

[B30] Holt S Reeder B Wilson M Harvey S Morrow JD Roberts LJ 2nd Moore K Increased lipid peroxidation in patients with rhabdomyolysis Lancet 1999 353 1241 10217088 10.1016/S0140-6736(98)05768-7

[B31] Reeder BJ Wilson MT The effects of pH on the mechanism of hydrogen peroxide and lipid hydroperoxide consumption by myoglobin: a role for the protonated ferryl species Free Radic Biol Med 2001 30 1311 1318 10.1016/S0891-5849(01)00534-2 11368929

[B32] Plotnikov EY Chupyrkina AA Pevzner IB Isaev NK Zorov DB Myoglobin causes oxidative stress, increase of NO production and dysfunction of kidney's mitochondria Biochim Biophys Acta 2009 1792 796 803 10.1016/j.bbadis.2009.06.005 19545623

[B33] Khan FY Rhabdomyolysis: a review of the literature Neth J Med 2009 67 272 283 19841484

[B34] Chen-Levy Z Wener MH Toivola B Daum P Reyes M Fine JS Factors affecting urinary myoglobin stability in vitro Am J Clin Pathol 2005 123 432 438 10.1309/9AQ62FR265ER3E2W 15716240

[B35] Premru V Kovač J Ponikvar R Use of myoglobin as a marker and predictor in myoglobinuric acute kidney injury Ther Apher Dial 2013 17 391 395 10.1111/1744-9987.12084 23931877

[B36] El-Abdellati E Eyselbergs M Sirimsi H Hoof VV Wouters K Verbrugghe W Jorens PG An observational study on rhabdomyolysis in the intensive care unit, Exploring its risk factors and main complication: acute kidney injury Ann Intensive Care 2013 3 8 10.1186/2110-5820-3-8 23497406 PMC3614462

[B37] Huerta-Alardín AL Varon J Marik PE Bench-to-bedside review: Rhabdomyolysis – an overview for clinicians Crit Care 2005 9 158 169 10.1186/cc3221 15774072 PMC1175909

[B38] Malik GH Rhabdomyolysis and myoglobin-induced acute renal failure Saudi J Kidney Dis Transpl 1998 9 273 284 18408300

[B39] KDIGO Clinical Practice Guideline for Acute Kidney Injury[http://www.kidney-international.org]

[B40] Finfer S Liu B Taylor C Bellomo R Billot L Cook D Du B McArthur C Myburgh J SAFE TRIPS Investigators Resuscitation fluid use in critically ill adults: an international cross-sectional study in 391 intensive care units Crit Care 2010 14 R185 10.1186/cc9293 20950434 PMC3219291

[B41] Myburgh JA Mythen MG Resuscitation fluids N Engl J Med 2013 369 1243 1251 10.1056/NEJMra1208627 24066745

[B42] Petejova N Martinek A Acute kidney injury following acute pancreatitis: a review Biomed Pap Med 2013 157 105 113 10.5507/bp.2013.048 23774848

[B43] Zager RA Foerder C Bredl C The influence of mannitol on myoglobinuric acute renal failure: functional, biochemical, and morphological assessments J Am Soc Nephrol 1991 2 848 855 1751788 10.1681/ASN.V24848

[B44] Bragadottir G Redfors B Ricksten SE Mannitol increases renal blood flow and maintains filtration fraction and oxygenation in postoperative acute kidney injury: a prospective interventional study Crit Care 2012 16 R159 10.1186/cc11480 22901953 PMC3580749

[B45] Brown CV Rhee P Chan L Evans K Demetriades D Velmahos GC Preventing renal failure in patients with rhabdomyolysis: do bicarbonate and mannitol make a difference? J Trauma 2004 56 1191 1196 10.1097/01.TA.0000130761.78627.10 15211124

[B46] Ouellet M Percival MD Mechanism of acetaminophen inhibition of cyclooxygenase isoforms Arch Biochem Biophys 2001 387 273 280 10.1006/abbi.2000.2232 11370851

[B47] Boutaud O Aronoff DM Richardson JH Marnett LJ Oates JA Determinants of the cellular specificity of acetaminophen as an inhibitor of prostaglandin H(2) synthases Proc Natl Acad Sci U S A 2002 99 7130 7135 10.1073/pnas.102588199 12011469 PMC124540

[B48] Bellomo R Kellum JA Ronco C Acute kidney injury Lancet 2012 380 756 766 10.1016/S0140-6736(11)61454-2 22617274

[B49] Heyne N Guthoff M Krieger J Haap M Häring HU High cut-off renal replacement therapy for removal of myoglobin in severe rhabdomyolysis and acute kidney injury: a case series Nephron Clin Pract 2012 121 159 164 10.1159/000343564 23327834

[B50] Tang W Chen Z Wu W Qiu H Bo H Zhang L Fu P Renal protective effects of early continuous venovenous hemofiltration in rhabdomyolysis: improved renal mitochondrial dysfunction and inhibited apoptosis Artif Organs 2013 37 390 400 10.1111/j.1525-1594.2012.01574.x 23441644

[B51] Amyot SL Leblanc M Thibeault Y Geadah D Cardinal J Myoglobin clearance and removal during continuous venovenous hemofiltration Intensive Care Med 1999 25 1169 1172 10.1007/s001340051031 10551978

[B52] Sorrentino SA Kielstein JT Lukasz A Sorrentino JN Gohrbandt B Haller H Schmidt BM High permeability dialysis membrane allows effective removal of myoglobin in acute kidney injury resulting from rhabdomyolysis Crit Care Med 2011 39 184 186 10.1097/CCM.0b013e3181feb7f0 21057310

[B53] Naka T Jones D Baldwin I Fealy N Bates S Goehl H Morgera S Neumayer HH Bellomo R Myoglobin clearance by super high-flux hemofiltration in a case of severe rhabdomyolysis: a case report Crit Care 2005 9 R90 R95 10.1186/cc3034 15774055 PMC1175920

[B54] Premru V Kovač J Buturović-Ponikvar J Ponikvar R High cut-off membrane hemodiafiltration in myoglobinuric acute renal failure: a case series Ther Apher Dial 2011 15 287 291 10.1111/j.1744-9987.2011.00953.x 21624078

[B55] Swaroop R Zabaneh R Parimoo N Plasmapheresis in a patient with rhabdomyolysis: a case report Cases J 2009 2 8138 10.4076/1757-1626-2-8138 19918458 PMC2769408

[B56] Ronco C Extracorporeal therapies in acute rhabdomyolysis and myoglobin clearance Crit Care 2005 9 141 142 10.1186/cc3055 15774064 PMC1175933

